# Toothache

**Published:** 1899-05

**Authors:** D. D. Atkinson


### Article VI.

### "TOOTHACHE."

D. D. ATKINSON, D. D. S.

People often ask how it is that a tooth can ache after the nerve has been killed; any so frequently is. this inquiry that the inference must be that very many people are under the impression that all toothache has its seat in a nerve contained within the tooth, and that this being destroyed, ought to forever end the possibility of pain in that particular tooth. That this idea is entirely erroneous is apparent to every dentist, but that it does prevail, even among otherwise intelligent people, cannot be denied.

It is the purpose of this brief article to point out some of the causes of toothache in teeth which have either living or dead pulps.

Every tooth has in its center a natural cavity conforming in shade to the outlines of the crown thereof, with canals leading to the end of each root. In this cavity is contained a living pulp (if the tooth be impaired), commonly known as the nerve. It consists of blood-vessels and nerve-fibres, connected with the vascular system, and nerve-centers through a small aperture at the end of each root or fang.

Now, so long as this pulp is not disturbed by external interference, its functions will very likely be performed, and all will be well; but when decay attacks the tooth from the outside (and this is mentioned because it is the most frequent cause of toothache), and makes so much progress that this delicate pulp, which has heretofore been protected by its solid walls, is exposed to the irritating effect of outside agencies, it frequently becomes the seat of inflammation, characterized by intense pain, which by reflex action may be transmitted to other teeth, or may be confined to only the affected tooth. An application of the proper remedies will relieve the pain and, if desired, kill the nerve. This, for a period, will send the pain from the offending tooth, but if the pulp cavity and the canals along the roots are not properly treated and filled, a recurrence of pain will be almost sure to follow in a short time, but from a different cause.

In the living pulp the increased flow of blood pressing against the unyielding cavity wall had caused the pain. When the pulp has been devitalized, and left to decompose within the tooth, it becomes a seat of infection, charged with poisonous gases and micro-organisms. These will find their way along the root canal, and make an exit through the small opening at the end of the root, and be deposited at the apex of the bony socket which contains the roots of the tooth. Violent inflammation will immediately follow, growing more intense, unless relief is afforded, until it terminates in alveolar abscess, and is discharged in the form of pus somewhere, usually on the surface of the gum, all the time being characterized by intense soreness to the touch. A tooth which, having its "nerve" killed, has been properly disinfected and filled, nearly always enjoys immunity from the condition above described, because there is no place for the lodgment of microorganisms. However, a tooth whose "nerve" is dead may remain for years inert, and yet might, upon some slight provocation, throw the patient into all the agonies of acute abscess. Without going further into the causes which lead to toothache, it is hoped this article has demonstrated how teeth whose "nerves" are killed can be the seat of that malady, as well as those whose "nerves" are intact.—*Information.*

